# Current situation in Europe : different prospective

**DOI:** 10.1192/j.eurpsy.2025.219

**Published:** 2025-08-26

**Authors:** I. Tarricone

**Affiliations:** University of Bologna, Bologna, Italy

## Abstract

**Abstract:**

Introduction. Previous evidence showed significant discrepancies in psychiatric services utilization between migrants and reference populations in Europe. An important barrier for migrants to Europe is the lack of adequate legal entitlements. Sometimes these barriers are mistakenly attributed to cultural differences and misunderstandings because the term **‘**culture’ may be used as a putative politically correct expression reifying social differences and neglecting discrimination. Our study aims were to evaluate incidence and characteristics of psychiatric hospitalizations of migrant patients compared with reference populations and to assess how the COVID-19 pandemic affected admissions. Methods. All patients admitted to the psychiatric ward **“**SPDC-Malpighi **”** of the Bologna Mental Health Department, Italy, from 01/01/2018 to 31/12/2020 were included. Differences in sociodemographic and clinical characteristics were tested by migrant status. Incidence rate ratios of hospital admissions by migrant status were estimated via Poisson regression considering population-at-risk, gender, and age-group. Results. Migrants had higher hospitalization rates due to any psychiatric disorder (IRR=1.16). The risk was especially pronounced among women (IRR=1.25) and within the youngest age-group (IRR=3.24). Young migrants had also a greater risk of compulsory admission (IRR=3.77). Regarding admissions due to a specific diagnosis, we found relevant differences in hospitalization rates for psychosis, mood disorders, and personality disorders. Finally, migrants were more likely to be admitted via the Emergency Department and less likely to be referred from a specialist. Discussion. During the year of pandemic (2020) we observed an increase in the proportion of migrants admitted voluntarily or compulsorily. Migrants, especially those from the youngest age-group, had higher hospitalization rates for any disorder. Younger migrants were also at higher risk of compulsory treatment. The distribution of psychiatric admissions during the pandemic period seemed to have further increased discrepancies in mental healthcare needs and provision between migrants and the reference population. Tailored interventions and policies are urgently needed to address this issue. Keywords Psychiatric admissions · Migrants · Pandemic · Compulsory treatments References: Tarricone I, D’Andrea G, Galatolo M, Carloni AL, Descovich C, Muratori R; Bo-East Psychiatric Admissions Study Group. Psychiatric Admission Among Migrants Before and During Pandemic: a Retrospective Study in Acute Psychiatric Ward in Bologna, Italy. J Immigr Minor Health. 2023 Jun;25(3):507-521. doi: 10.1007/s10903-023-01464-7. Epub 2023 Mar 23. PMID: 36952152; PMCID: PMC10034892.

**Disclosure of Interest:**

None Declared

